# Derivation of an amino acid similarity matrix for peptide:MHC binding and its application as a Bayesian prior

**DOI:** 10.1186/1471-2105-10-394

**Published:** 2009-11-30

**Authors:** Yohan Kim, John Sidney, Clemencia Pinilla, Alessandro Sette, Bjoern Peters

**Affiliations:** 1 Division of Vaccine Discovery, La Jolla Institute for Allergy and Immunology, La Jolla, California, USA; 2 Immunology, Torrey Pines Institute for Molecular Studies, San Diego, California, USA

## Abstract

**Background:**

Experts in peptide:MHC binding studies are often able to estimate the impact of a single residue substitution based on a heuristic understanding of amino acid similarity in an experimental context. Our aim is to quantify this measure of similarity to improve peptide:MHC binding prediction methods. This should help compensate for holes and bias in the sequence space coverage of existing peptide binding datasets.

**Results:**

Here, a novel amino acid similarity matrix (PMBEC) is directly derived from the binding affinity data of combinatorial peptide mixtures. Like BLOSUM62, this matrix captures well-known physicochemical properties of amino acid residues. However, PMBEC differs markedly from existing matrices in cases where residue substitution involves a reversal of electrostatic charge. To demonstrate its usefulness, we have developed a new peptide:MHC class I binding prediction method, using the matrix as a Bayesian prior. We show that the new method can compensate for missing information on specific residues in the training data. We also carried out a large-scale benchmark, and its results indicate that prediction performance of the new method is comparable to that of the best neural network based approaches for peptide:MHC class I binding.

**Conclusion:**

A novel amino acid similarity matrix has been derived for peptide:MHC binding interactions. One prominent feature of the matrix is that it disfavors substitution of residues with opposite charges. Given that the matrix was derived from experimentally determined peptide:MHC binding affinity measurements, this feature is likely shared by all peptide:protein interactions. In addition, we have demonstrated the usefulness of the matrix as a Bayesian prior in an improved scoring-matrix based peptide:MHC class I prediction method. A software implementation of the method is available at: http://www.mhc-pathway.net/smmpmbec.

## Background

Amino acid similarity matrices define a quantitative measure of likeness between each of the 20 canonical amino acids. They are utilized throughout computational biology in areas such as phylogenetics, protein structure modeling, and prediction of protein ligand interactions. Depending on the application, different measures of similarity are appropriate. For example, the commonly used PAM and BLOSUM matrices [1,2] have been built based on the frequencies of amino acid substitutions observed in aligned protein sequences. This measure, routinely used in programs such as BLAST [3], represents both evolutionary and functional similarity between amino acids.

Our group has been interested in amino acid similarity in the context of peptides binding to proteins. Given binding data for several peptide ligands, the challenge is to predict the affinity of any peptide of arbitrary sequence. Our specific interest is in peptide binding to proteins involved in antigen processing and presentation, such as the TAP transporter [4,5] and MHC molecules. In recent large-scale benchmark studies, the best performing prediction method for peptide:MHC class I binding is the NetMHC artificial neural network, outperforming linear methods such as SMM [6,7]. NetMHC is trained using a BLOSUM matrix based encoding of peptide sequences [8-13]. This provides the neural network with information on amino acid similarity, and allows it to predict the impact of residues on binding that are not represented in the training set.

In this study, we tested the hypothesis that amino acid similarity in the context of peptide binding to MHC molecules is distinct from previously defined metrics. We further examined if this similarity measure can be used to improve peptide:MHC binding predictions, and if incorporating it into the SMM approach can close the gap in prediction quality to NetMHC.

## Results

### Combinatorial peptide library binding affinity data

A library of combinatorial peptide mixtures was used to measure the binding affinity contribution of each residue in a 9-mer peptide to an MHC molecule. The library contains mixtures of 9-mer peptides all sharing the same residue type at one position, while the remaining positions are allowed to sample all residue types. For instance, 'XAXXXXXXX' represents a mixture of peptides with an Alanine at position P2, and any one of the possible residues at the remaining positions. A total of 180 mixtures covering 20 residue types in all positions of a 9-mer peptide were synthesized, and tested for binding to 24 MHC class I molecules listed in the methods section. Thus, a total of 180 × 24 = 4320 binding affinities in terms of IC_50 _values were measured (Additional file 1: Dataset S1). We then transformed these values to approximate a relative binding energy contribution of an amino acid *aa *at peptide position *pos *for a given *MHC *molecule:

### Building the peptide:MHC binding energy covariance (PMBEC) matrix

**Figure 1:**
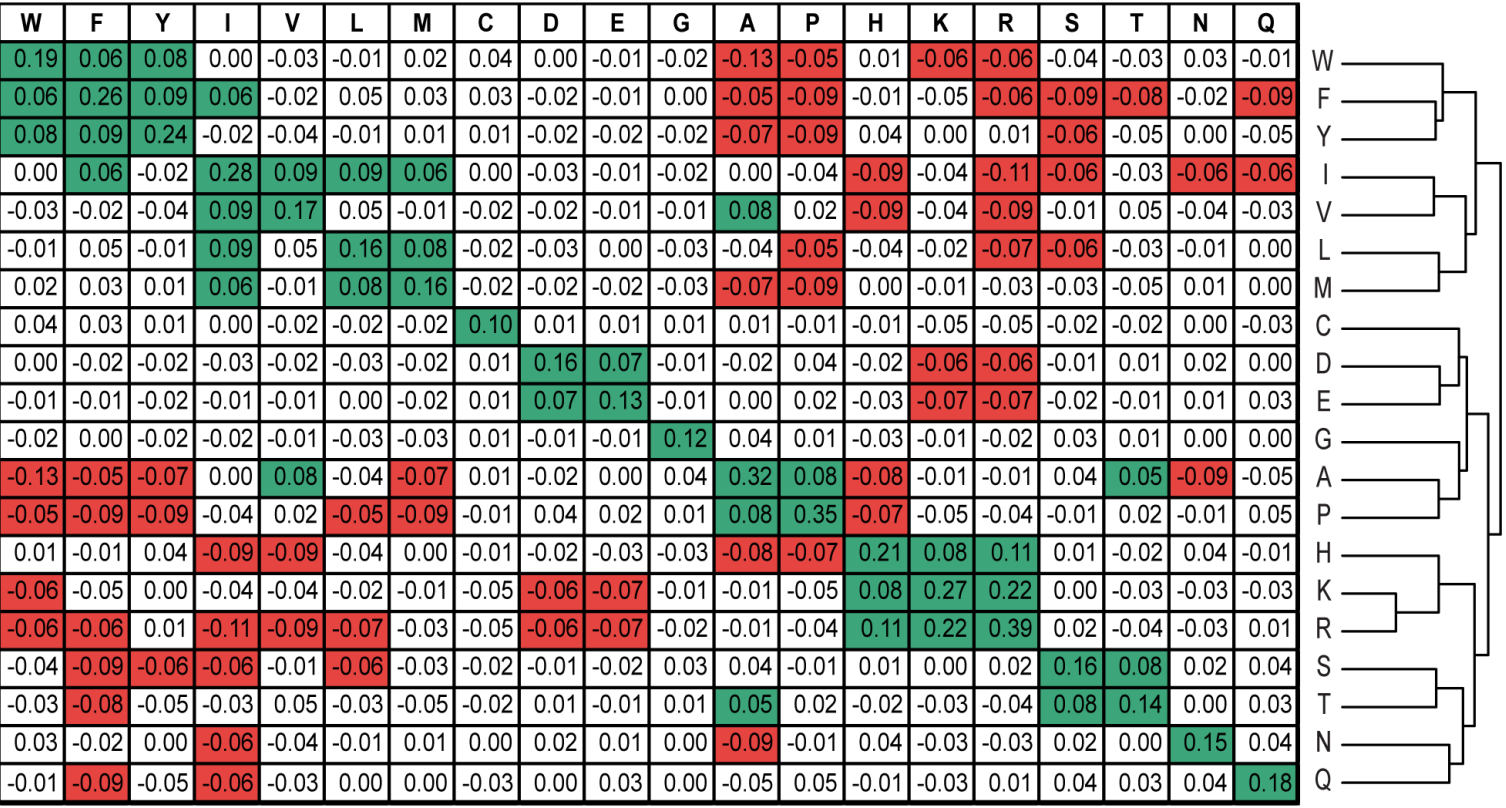
**The peptide:MHC binding energy covariance (PMBEC) matrix**. The 20 amino acid residues are shown at the top and right. Each matrix entry corresponds to the covariance in peptide:MHC binding energies between two residues. Values greater than 0.05 indicate similarity between residues, and are colored green. Values less than -0.05 indicate dissimilarity between residues, and are colored red. Note that the diagonal values are the residue specific statistical variances (defined as the average squared values), which indicate how much the binding energies associated with the residue varies over all alleles and positions. Cysteine (C), Glycine (G), Asparagine (N), and Glutamine (Q) are relative outliers because they have no partner residue with absolute covariance > 0.05. Agglomerative clustering with complete linkage was used to group the amino acid residues, corresponding to ordering the matrix rows and columns. The distance measure between two residues aa and aa' used for clustering is (K - PMBEC(aa, aa')), where K is the maximum value in the PMBEC matrix. The resulting dendrogram on the right provides a classification of amino acids which largely corresponds to classical groupings of amino acids by physicochemical properties.

To quantify how similar two amino acids *aa *and *aa' *are in the context of peptide:MHC binding, covariance of their relative binding energy contributions, *ΔE*_*aa*, *pos*, *MHC *_and *ΔE*_*aa*', *pos*, *MHC*_, was calculated as follows:

Variables *ΔE*_*aa *_and *ΔE*_*aa*' _are averages over all positions and MHC molecules for amino acids *aa *and *aa'*, respectively. These covariance values define the **Peptide:MHC Binding Energy Covariance (PMBEC) Matrix**. A positive covariance between two residues indicates that, on average, they contribute similarly to binding free energy in different environments. Conversely, a negative covariance indicates that when one residue contributes favorably to binding, the other contributes unfavorably. Figure 1 shows the PMBEC matrix, which is symmetric and has dimensions of 20 × 20. The matrix is also provided as a text file (Additional file 2: Dataset S2). Clustering amino acids based on their covariances resulted in amino acid groupings similar to their classically known physicochemical properties: aromatic (W, F, and Y); hydrophobic (L, I, V, and M); acidic (D and E); basic (R, H, and K); small (G, A, and P); small and polar (S and T); and polar (N and Q). This indicated that the PMBEC matrix was in agreement with existing heuristic groupings of amino acids.

### Comparing the PMBEC matrix to previously established measures of amino acid similarity

Numerous amino acid similarity matrices have been published to date. Some of more prominent matrices such as BLOSUM series have been, in fact, used in peptide:MHC binding predictions to represent peptide sequences, despite fundamental differences in context of their use [13]. Apparent success of their use indicates that it is worthwhile to investigate how similar the PMBEC matrix is to these matrices.

Toward this end, we retrieved all 135 matrices out of 141 without missing entries from the AA Index Database [14], centered these matrices, and calculated their Pearson's correlation coefficients with respect to the PMBEC matrix (See Methods for details). Table 1 lists the 10 matrices from the AA Index database that are most similar to the PMBEC matrix. In the table, BLOSUM50 has the highest correlation coefficient of 0.64, followed by OPTIMA, Johnson & Overington, BLOSUM62, and BLOSUM80, among others. OPTIMA was derived by optimizing a matrix to distinguish between remote homologues and non-homologues. The Johnson & Overington matrix was derived from a tabulation of amino acid substitutions observed in sequences that were aligned using three-dimensional protein structures. Furthermore, BLOSUM-X matrices with increasing X (i.e. based on alignments with increasing sequence homology) decrease in similarity to PMBEC. Taken together, these observations indicate that the PMBEC matrix most strongly resembles amino acid similarity matrices that were assembled based on alignment of evolutionarily distant protein sequences.

**Table 1:** Ten most similar matrices to PMBEC out of all complete matrices from the AA Index Database.

Matrix Identifier	Cor.|PMBEC	Cor.|BLOSUM62	Description
HENS920104	0.64	0.96	BLOSUM50 [1]
KANM000101	0.63	0.96	OPTIMA [23]
JOHM930101	0.63	0.87	Johnson & Overington [24]
HENS920102	0.62	1.00	BLOSUM62 [1]
KOSJ950115	0.62	0.81	Koshi & Goldstein [25]
HENS920103	0.61	0.97	BLOSUM80 [1]
OVEJ920105	0.61	0.69	Overington et al. [26]
BENS940103	0.61	0.93	Benner et al. [27]
VOGG950101	0.61	0.93	Vogt et al. [28]
GONG920101	0.61	0.93	Gonnet et al. [29]

From the AA Index Database [14], all complete 135 amino acid similarity matrices out of 141 were downloaded and compared to PMBEC based on their Pearson's correlation coefficients in the second column. See Methods for details. Also shown are correlation coefficients with respect to BLOSUM62 in the third column.

Another observation one can make about the matrices in the table is that the PMBEC matrix falls outside the cluster of matrices that most resembles it. Specifically, in contrast to a correlation coefficient of 0.64 between PMBEC and BLOSUM50, seven out of ten matrices in the table have more than 0.93 with respect to BLOSUM50. This further adds to evidence that the PMBEC matrix is novel.

**Figure 2:**
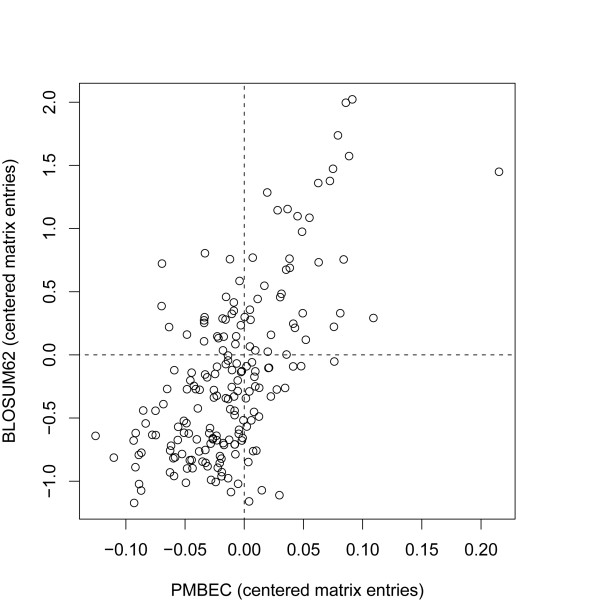
**A scatter plot of non-diagonal elements of PMBEC versus those of BLOSUM62**. The two matrices were centered as described in the method section.

To further characterize the PMBEC matrix, we carried out additional comparisons with BLOSUM62. Figure 2 plots correlation of matrix elements between BLOSUM62 and PMBEC with a correlation of 0.62. BLOSUM62 was chosen because it is representative of the matrices in Table 1; it has high correlations (i.e. > 0.90) with most of the matrices in the table including BLOSUM50. Also, it is widely used in programs such as BLAST.

**Figure 3:**
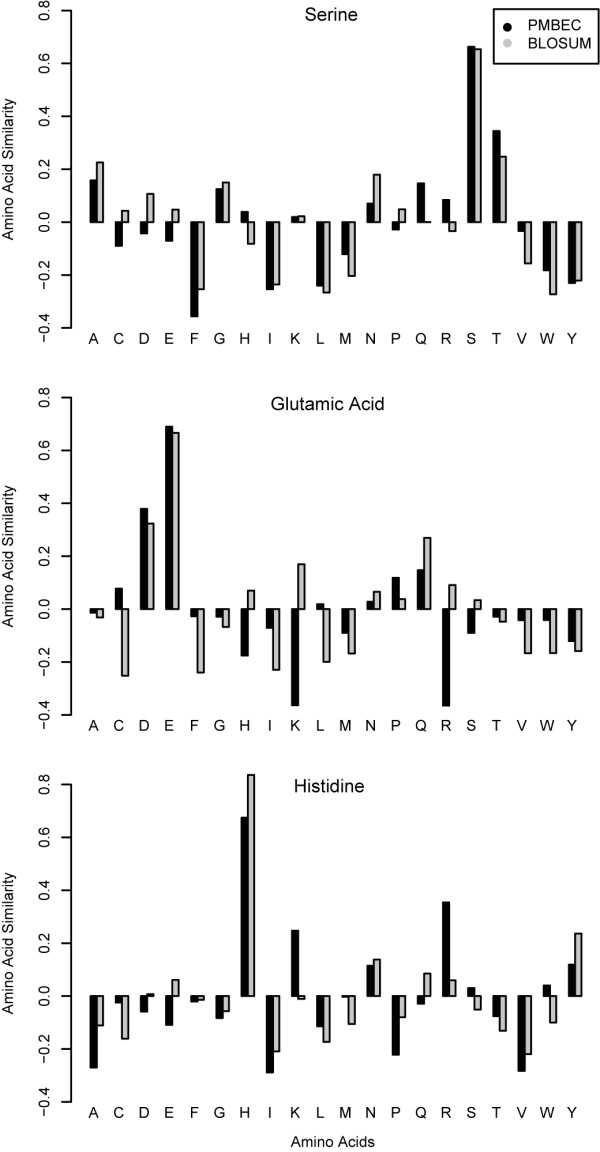
**Comparisons of amino acid similarity profiles of PMBEC and BLOSUM62**. Each amino acid profile of 20 elements was normalized to a length of 1.0 with zero mean to allow direct comparison between the two matrices. Serine-specific amino acid similarity profiles of the two matrices share a high correlation. Glutamic Acid-specific ones, however, significantly differ for the substitutions involving charged residues: (Glutamic Acid (E) -> Lysine (K)) and (Glutamic Acid (E) -> Arginine (R)).

Accordingly, Figure 3 depicts detailed comparisons between the matrices for three residues: Serine, Glutamic acid, and Histidine. While Serine-specific amino acid similarity profiles of BLOSUM62 and PMBEC share a high correlation of 0.91, those of Glutamic acid share a relatively low correlation of 0.61. A closer look at the substitution profiles indicates that PMBEC highly disfavors substitution of oppositely charged residues, Glutamic acid (E) with Arginine (R), while BLOSUM62 is neutral. Similarly, PMBEC disfavors substitution of Glutamic acid (E) with Lysine (K), while BLOSUM62 favors this slightly. For Histidine specific profiles, we see that substitution of similarly charged residues, Histidine (H) and Lysine (K)/Arginine (R), are favored by PMBEC while BLOSUM considers them essentially neutral. Furthermore, five amino acids with the lowest correlation coefficients include Glutamic acid (E), Cysteine (C), Asparagine (N), Lysine (K), and Aspartic acid (D) (data not shown). Taken together, these observations indicate that differences between BLOSUM62 and PMBEC are most pronounced where charged residues are involved.

In the following sections, a novel peptide:MHC binding prediction method SMM^PMBEC ^is introduced, and its benefits are illustrated. SMM^PMBEC ^improves upon the original SMM approach [15] by using PMBEC as a Bayesian prior. The details of how a Bayesian prior used in the SMM approach is described in the Methods section.

### SMM^PMBEC ^can compensate for the absence of important subset of binding data

Peptide binding affinity measurements available for MHC class I molecules can vary from less than a hundred to thousands for each allele. Furthermore, peptide sequence space coverage in the binding data can be limited even for alleles with large data sets, due to biases when selecting peptides for binding measurements. Here, we examined how well SMM^PMBEC ^addresses the problem of missing data. We then compared its performance to that of SMM^BLOSUM^, which uses BLOSUM62 as a prior.

**Figure 4:**
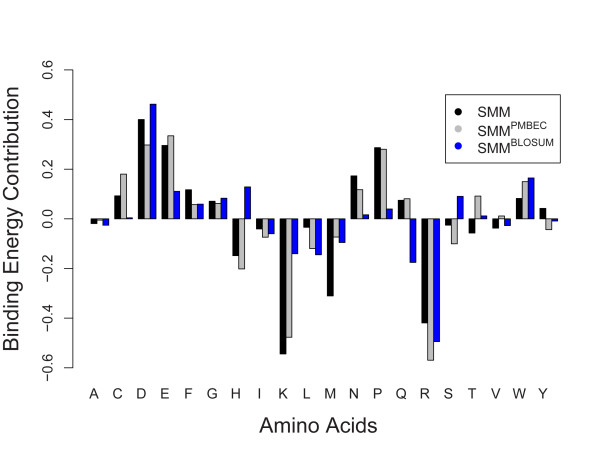
**Comparison of binding contributions of 20 amino acids at position 1 of the scoring matrices generated by SMM, SMM^PMBEC^, and SMM^BLOSUM^**. SMM was trained on the 9-mer peptide binding data set (total of 1869 data points) for HLA A*3101, yielding a single scoring matrix with dimensions 20 × 9, where the rows represent 20 residues while the columns represent 9 positions of a peptide. The scoring matrix generated by SMM serves as a reference point when binding data is well covered. SMM^PMBEC ^and SMM^BLOSUM^, on the other hand, were trained on the 20 derived data sets, each one lacking peptides containing a residue at position 1. The figure plots the scoring matrix values for the residue specified on the x-axis in the second column of the scoring matrix of SMM alongside corresponding elements from the 20 scoring matrices of SMM^PMBEC ^and SMM^BLOSUM^.

To establish a reference point, SMM was trained on the 1869 peptide binding affinity measurements available for HLA A*3101 [7], resulting in a 20 × 9 scoring matrix. Because SMM has been shown to train an accurate model for this allele and because of the large amount of data available, we assumed that the scoring matrix closely approximated the 'true' binding specificity. The SMM matrix entries for peptide position P1 for 20 residues are shown as black bars in Figure 4. According to this scoring matrix, Lysine, Arginine, Methionine, and Histidine (K, R, M, and H) contribute the most favorable binding energies (i.e. negative values); while Aspartic acid, Glutamic acid, Proline, and Asparagine (D, E, P, and N) contribute the least favorable binding energies (i.e. positive values).

We then derived a subset from the original binding data for HLA A*3101 such that all peptides with an Alanine at P1 were excluded. When SMM was trained on this subset, its scoring matrix gave a value of zero at P1 for residue Alanine since SMM had no information on it available from the training data. When training SMM^PMBEC ^on this subset, its scoring matrix entry for Alanine at P1 gave a non-zero value. This step was repeated for the remaining 19 residues, and their corresponding scoring matrix entry values are shown as gray bars in Figure 4. In the figure, three out of four residues with the most favorable binding energy contribution - Arginine, Lysine, Histidine (R, K, and H) - are shared between SMM and SMM^PMBEC^. Similarly, three out of four residues with the least favorable binding energy contribution - Glutamic acid, Aspartic acid, and Proline (E, D, and P) - are shared between the two methods. The binding energy profiles of SMM and SMM^PMBEC ^had a Pearson's correlation value of 0.92. Thus, these observations indicate that SMM^PMBEC ^can infer binding energies of missing residues from those present in the binding data.

When we repeated this exercise for SMM^BLOSUM^, resulting in a binding energy profile shown in blue in Figure 4, we saw that SMM^BLOSUM ^can also infer binding energies of missing residues (r = 0.69). However, approximated binding energies for Lysine (K) and Histidine (H) are sharply different between SMM^PMBEC ^and SMM^BLOSUM^. The obvious benefit of using SMM^PMBEC ^for HLA A*3101 binding data set is by no means true for all other data sets. However, this example highlights what can be achieved with a properly tuned amino acid similarity matrix.

### SMM^PMBEC ^consistently outperforms SMM over a wide range of training data set sizes

**Figure 5:**
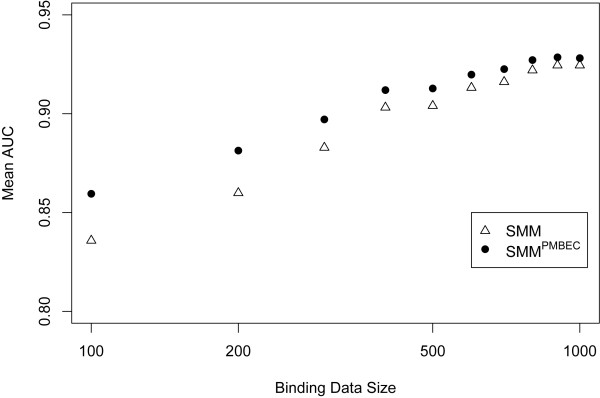
**Prediction performances of SMM^PMBEC ^and SMM, trained on data sets with variable amounts of peptide binding data**. For each data set size, 20 data sets were randomly drawn from the peptide binding data of HLA A*1101. The average AUC (Area-Under-Curve) values of the two prediction methods are plotted as a function of the dataset size.

To further compare SMM^PMBEC ^and SMM, we tested whether SMM^PMBEC ^can outperform SMM over a wide range of training data sizes. Figure 5 shows averaged prediction performances of SMM^PMBEC ^and SMM trained on randomly sampled data sets for each size, all sampled from the binding data of HLA A*1101. The allele HLA A*1101 was chosen mainly because of its relatively large data size, thereby allowing data sampling with a wide range of data sizes. Five-fold cross validation was used for each data set sampled (See Methods). The figure shows that for small training data sets where a lack of sequence coverage is more likely, SMM^PMBEC ^has statistically significant performance improvements over that of SMM. For example, for the data set size of 100 measurements, averaged prediction performances for SMM^PMBEC ^and SMM are 0.860 and 0.836 respectively (p-value = 2.0E-05).

### Application of SMM^PMBEC ^in peptide:MHC-I binding predictions

Motivated by the promising performance of SMM^PMBEC^, we carried out a large-scale performance evaluations as described in [7], where neural network based NetMHC [13] was shown to be the best performing method, followed by SMM, which uses scoring matrices [15]. Prediction performances of SMM, SMM^PMBEC^, and NetMHC are shown in Table 2 for 46 alleles.

**Table 2:** Prediction performances as measured by AUC values of ROC curves.

MHC	Data Size	SMM	SMM^PMBEC^	NetMHC
H-2_Db	303	0.909	0.901	0.933
H-2_Dd	85	0.813	0.837	0.925
H-2_Kb	223	0.811	0.833	0.850
H-2_Kd	176	0.928	0.931	0.939
H-2_Kk	164	0.772	0.793	0.790
H-2_Ld	102	0.932	0.942	0.977
HLA_A-0101	1157	0.977	0.977	0.982
HLA_A-0201	3089	0.946	0.946	0.957
HLA_A-0202	1447	0.898	0.899	0.900
HLA_A-0203	1443	0.916	0.916	0.921
HLA_A-0206	1437	0.913	0.916	0.927
HLA_A-0301	2094	0.927	0.928	0.937
HLA_A-1101	1985	0.938	0.939	0.951
HLA_A-2301	104	0.793	0.840	0.852
HLA_A-2402	197	0.803	0.801	0.825
HLA_A-2403	254	0.919	0.932	0.918
HLA_A-2601	672	0.916	0.924	0.956
HLA_A-2902	160	0.912	0.916	0.935
HLA_A-3001	669	0.935	0.941	0.947
HLA_A-3002	92	0.878	0.830	0.744
HLA_A-3101	1869	0.925	0.925	0.928
HLA_A-3301	1140	0.923	0.925	0.915
HLA_A-6801	1141	0.885	0.885	0.883
HLA_A-6802	1434	0.899	0.899	0.899
HLA_A-6901	833	0.867	0.880	0.880
HLA_B-0702	1262	0.959	0.960	0.965
HLA_B-0801	708	0.932	0.956	0.955
HLA_B-1501	978	0.937	0.940	0.941
HLA_B-1801	118	0.881	0.880	0.838
HLA_B-2705	969	0.936	0.941	0.938
HLA_B-3501	736	0.883	0.889	0.875
HLA_B-4002	118	0.832	0.843	0.754
HLA_B-4402	119	0.731	0.739	0.778
HLA_B-4403	119	0.757	0.753	0.763
HLA_B-4501	114	0.825	0.866	0.862
HLA_B-5101	244	0.876	0.895	0.886
HLA_B-5301	254	0.889	0.885	0.899
HLA_B-5401	255	0.923	0.935	0.903
HLA_B-5701	59	0.826	0.843	0.826
HLA_B-5801	988	0.942	0.945	0.961
Mamu_A-01	525	0.855	0.854	0.861
Mamu_A-02	283	0.765	0.783	0.809
Mamu_A-11	468	0.883	0.894	0.894
Mamu_B-01	205	0.949	0.956	0.967
Mamu_B-17	300	0.934	0.943	0.954
Patr_B-0101	132	0.964	0.975	0.969

average AUC		0.887	0.894	0.897
t-test|SMM		NA	0.001	0.057
t-test|NetMHC		0.057	0.470	NA

For each MHC-specific binding data set, 5-fold cross validation was carried out using one of the four methods listed. Area-Under-Curve (AUCs) from the 5-fold cross validations are presented here. Also shown are p-values of average AUC values calculated using Student's t-test (2-tail, paired) with respect to the SMM and NetMHC prediction methods.

When looking at these measured prediction performances in Table 2, some precautions are in order. It has been observed earlier that accuracy of a predictor tends to increase with more training data [7]. Likewise, reliability of a predictor's accuracy also increases with more training data, because larger peptide sequence space is sampled. Thus, apparently high prediction accuracy of a predictor for Patr B*0101, for instance, should not be taken at its face value, because the predictor was trained on relatively small training data (132 data points).

With these precautions in mind, as shown in Table 2, SMM^PMBEC ^outperformed SMM in 39 out of 46 cases, and the average performance of SMM^PMBEC ^was higher than SMM (0.894 vs. 0.887 AUC values). The small but consistent improvements are statistically significant with p-value of 0.001 using Student's t-test (paired two tailed; the test assumes that AUC values over data sets follow a normal distribution). The same comparison for SMM^PMBEC ^and NetMHC indicated that their difference of average performances was not statistically significant (p-value = 0.47). Thus, the use of PMBEC as a Bayesian prior improves performance for SMM^PMBEC^, yielding a performance that is overall comparable to that of NetMHC. Given that NetMHC uses neural networks to represent a model of peptide:MHC binding specificity, it is noteworthy that the use of a simple scoring matrix coupled with a Bayesian prior can match its prediction performance.

A closer look at Table 2 reveals that the most significant performance improvements of SMM^PMBEC ^over SMM tend to come from alleles with smaller data sets (e.g. HLA A*2301, HLA B*4501). This is in agreement with what has been observed earlier that at smaller data size, the benefits of PMBEC as a Bayesian prior compensating for missing binding data are more apparent.

We have also carried out performance evaluations for SMM^BLOSUM ^(Additional file 3: Dataset S3). The difference between averaged prediction performances of SMM^BLOSUM ^and SMM^PMBEC ^was 0.0003, which is not statistically significant (p-value = 0.83). We attribute their similar performances mainly to a feature of SMM approach where prior is relied on less as more binding data become available for training. In fact, when prediction performance differences of SMM^PMBEC ^and SMM^BLOSUM ^are plotted against training data set sizes, a clear pattern emerges where larger training data sizes correlate with smaller performance differences (data not shown). About 80% of data sets are contained within ± 0.01 AUC of zero performance difference.

Of the remaining 20% of the data sets, we see that three MHCs with the highest performance differences favoring PMBEC have binding motifs with strong preference for Glutamic acid at the anchor residue position. (Of these three MHCs, HLA B*4403, followed by B*4002 and H-2 KK, has the largest AUC difference of 0.037.) This latter observation supports one of the main arguments made in the present work that amino acid similarities involving Glutamic acid are the most prominent difference between PMBEC and BLOSUM. This in turn suggests that those MHC's with Glutamic acid as anchor residues is where SMM^PMBEC^'s prediction performances will be superior to those of SMM^BLOSUM^.

## Discussion

We have derived a novel amino acid similarity matrix (PMBEC) for peptide:MHC class I binding. Rather than relying on sequence alignments, the matrix was derived from experimentally measured binding affinities of combinatorial peptide mixtures. The use of combinatorial peptide mixtures afforded us an unbiased sampling of peptide sequence space. In total, a panel of 24 MHC class I molecules was probed, corresponding to 4320 binding affinity measurements of individual residues in diverse contexts of both peptide ligand and receptor molecule. This approach can, therefore, directly evaluate amino acid similarities in the context of peptide:protein binding.

Once the PMBEC matrix was derived, its comparisons with all complete 135 amino acid similarity matrices taken from the AA Index Database have shown that the matrix is different from previously established amino acid similarity matrices. To determine where PMBEC most differs, detailed comparisons with BLOSUM62 (representative of those matrices most similar to PMBEC) have revealed that PMBEC considers pairs of amino acids with opposite charges to be very dissimilar, while BLOSUM62 considers them a neutral exchange. These differences are most likely due to different molecular consequences of substituting an amino acid in a protein, and in a peptide bound to MHC. Specifically, in protein sequences, most charged residues are on the surface, and a reversal of charge can often be tolerated well, as it preserves hydrophilicity. For peptide ligands, on the other hand, a reversal of charge is likely going to adversely affect their binding affinities.

Because of these peptide:protein specific features, we expect the PMBEC matrix will be useful in modelling peptide similarities in the context of immune recognition. In addition, we also expect that the matrix will be of use to those studying other types of peptide:protein interactions (e.g. SH3, PDZ, and WW protein domains). In hindsight, we are surprised that a novel amino acid similarity matrix such as PMBEC can still be discovered. Our work further underscores the importance of application-specific amino acid similarity matrices in computational biology, since molecular context determines which matrices are more meaningful.

In the context of predicting MHC class I binding peptides, we have shown that SMM^PMBEC ^- which uses PMBEC as a Bayesian prior - has a significantly better prediction performance than SMM. For those alleles with small data sets, performance improvements were more apparent, indicating that the use of PMBEC as a Bayesian prior is likely compensating for inadequate peptide sequence coverage. We have also shown that SMM^PMBEC^'s ability to infer 'true' binding energy contributions of intentionally excluded residues from others present in the binding data is limited. This is probably due to subtle differences of MHC molecule binding specificities that have so far not been appreciated. For comparison, a Bayesian prior based on BLOSUM62 also displays this property.

We have also shown that the average performance difference of SMM^PMBEC ^and NetMHC (the best performing method for peptide:MHC class I binding prediction according to recent benchmarks) is not statistically significant. Our results indicate that, at least for peptide:MHC class I binding predictions, scoring matrices can provide competitive prediction performances. This close prediction performances between a non-linear model and a linear one suggests that the limit of a linear model has been reached. From a practical standpoint, the key advantages of using a scoring matrix are that the model is easy to understand, interpret, and communicate.

Lastly, although the use of PMBEC improves prediction performance, the simplicity of the approach comes with a number of inherent limitations. One notable limitation is that the same set of amino acid similarity rules encoded in PMBEC is used for all peptide positions. Since there are position dependent influences observed for peptide:MHC binding motifs, such information may be used in the near future with more experimental data.

## Conclusion

PMBEC is a novel amino acid similarity matrix derived for peptide:MHC class I binding. One prominent feature of the matrix is that it disfavors substitutions of amino acids with opposite charges. This is likely a general feature of peptide:protein interactions. We have also demonstrated the usefulness of PMBEC in the context of peptide:MHC class I binding predictions, by using it as a Bayesian prior in a new prediction method SMM^PMBEC^. Results from a large-scale benchmark indicate that its prediction performance rivals that of one of the best performing methods in the field.

## Methods

### Positional scanning combinatorial peptide library and peptide synthesis

The combinatorial library was synthesized as previously described [16]. Each pool in the library contains 9-mer peptides with one fixed residue at a single position. With each of the 20 naturally occurring residues represented at each position along the 9-mer backbone, the entire library consisted of 180 peptide mixtures. Peptides utilized in screening studies were synthesized as described elsewhere [17], or purchased as crude material from Mimotopes (Minneapolis, MN/Clayton, Victoria, Australia), Pepscan Systems B.V. (Lelystad, Netherland) or A and A Labs (San Diego, CA). Peptides synthesized for use as radiolabeled ligands were synthesized by A and A Labs and purified to >95% homogeneity by reverse phase HPLC. Purity of these peptides was determined using analytical reverse-phase HPLC and amino acid analysis, sequencing, and/or mass spectrometry. Peptides were radiolabeled with the chloramine T method [18]. Lyophilized peptides were re-suspended at 4-20 mg/ml in 100% DMSO, then diluted to required concentrations in PBS +0.05% (v/v) nonidet P40 (Fluka Biochemika, Buchs, Switzerland).

### Target major histocompatibility complex (MHC) molecules for the generation of binding data using combinatorial peptide mixtures

For each one of the 24 MHC molecules, a scoring matrix was generated. The target MHC molecules came from four organisms: human (A*0201, A*6802, A*3201, A*3001, B*5802, B*5801, B*5401, B*5301, B*5101, B*3501, B*2705, B*1503, B*1501, B*0801, and B*0702); mouse (H-2 Kk, H-2 Kd, H-2 Dd, and H-2 Db); chimpanzee (Patr A*0401 and Patr A*0301); and macaque (Mamu B*08, Mamu B*03, and Mamu B*01). Binding data for human and mouse have been published in [19]; those for chimpanzee in [20]; and those for macaque have been submitted.

### MHC purification and peptide binding assays

MHC purification and quantitative binding assays based on the inhibition of binding of a high affinity radiolabeled ligand were performed essentially as described elsewhere [18-21]. In competition assays, each mixture or individual peptide was tested in 3 or more independent experiments for its capacity to inhibit the binding of the radiolabeled peptide. The concentration of peptide yielding 50% inhibition of the binding of the radiolabeled peptide was calculated. Under the conditions utilized, where the concentration of the labelled ligand is less than that of MHC molecule and IC_50 _is greater than the concentration of MHC molecule, the measured IC_50 _values are reasonable approximations of dissociation constant, KD.

### Comparing amino acid similarity matrices from different sources

An accurate comparison of amino acid matrices was important in this study because we wanted to determine whether the peptide:MHC binding covariance matrix (PMBEC) introduced in this work is significantly different from others. Toward this, amino acid similarity matrices were first centered [22] as shown for the matrix *A*,

where *H = I - J/n*, *I *is an identity matrix, *J *is a matrix composed of ones, and *n *is 20. Centering of matrices reduces the influence of data source dependent expected probabilities. Following this, Pearson's correlation coefficients of matrices with respect to PMBEC were calculated. Out of 210 unique elements for each symmetric 20 × 20 matrix, 190 non-diagonal elements were considered for the calculation of correlation since we are interested in relationships involving two *different *residues.

### Application of PMBEC as a Bayesian prior

We have previously established the SMM prediction method [15], which models the peptide binding specificity for a given MHC allele as a scoring matrix. Each matrix entry corresponds to the predicted binding energy contribution of a residue in a fixed length peptide. The matrix is determined by minimizing the difference between predicted and measured binding affinities in combination with a regularization term, which serves to push any entry in the scoring matrix toward zero for which no clear contribution to binding can be determined from the training data. This approach has been shown to vastly outperform other scoring matrix based predictions such as ARB and Rankpep [6,7], but it is outperformed by NetMHC, which uses artificial neural networks and amino acid similarity information.

Motivated by this observation, we reasoned that prediction performances of SMM can also be improved by incorporating amino acid similarity information. We reframed SMM from a Bayesian viewpoint, and assigned prior probabilities to scoring matrices by assuming that their matrix entries follow the multivariate normal distribution defined by the covariance values in PMBEC. The minimization of differences between measured and predicted affinities corresponds to maximizing the likelihood of observing the measured binding affinities for a given scoring matrix. It can be shown that the regularization term in the original SMM approach corresponds to the use of a Bayesian prior that assumes a multivariate normal distribution for the scoring matrix but assumes no correlation between different amino acids. In essence, the use of this Bayesian prior now favors scoring matrices that reflect amino acid similarity information encoded in PMBEC. Details of the derivation are shown in the following section.

From here on, SMM refers to the original version of the method, where an identity matrix is used in a prior. Likewise, SMM^PMBEC ^and SMM^BLOSUM ^use PMBEC and BLOSUM62 matrices in their priors, respectively.

### Implementation of Bayesian prior into SMM

To build a model of binding specificity of an MHC molecule for 9-mer peptides, a scoring matrix is trained, given *N *binding affinity measurements, *b*_*m*_*(Nx1)*. The scoring matrix is represented as a vector including an offset variable, yielding a total of 20 × 9 + 1 = 181 rows: *w(181x1)*. Peptide sequences are represented using a sparse encoding scheme, where a binary vector of length 20 is associated with each residue position. Thus, *N *9-mer peptides can be represented by a matrix, *H(Nx181)*. Binding predictions are carried out by a matrix multiplication *H *and *w*, resulting in a vector of predicted binding affinities for the peptides.

Assuming that errors in binding affinity measurements, *b*_*m*_, are normally distributed with variance, *δ*^2^, and zero mean, the likelihood of the binding measurements given a scoring matrix, *w*, is:

Assuming that the columns of the scoring matrix, *w*, follow the multivariate normal distribution specified by the covariance matrix, *C*, the prior is:

From this Bayesian viewpoint, it can be seen that the SMM approach uses an identity matrix for the covariance matrix, *C*, in a prior. Using Bayes' theorem, the posterior can now be defined as:

By maximizing the posterior, the model, *w*, is optimized to best correlate with the binding measurements, *b*_*m*_, with respect to the prior. To solve for *w*, both sides of the equation are log transformed and then multiplied by the experimental error, *δ*^2^, to yield:

After differentiating the equation and solving for *w*, it can be shown that the following equation is an analytical solution for *w *that minimizes the above equation:

where λ = *δ*^2 ^now serves as a scaling factor balancing the influences of *p(b*_*m*_|*w) *and *p(w)*. Put it differently, λ determines how much influence the covariance matrix has at each residue position. The optimal λ is determined by minimizing the cross-validated distance shown below:

Here, the binding data was split into five parts where one part is labelled 'blind' and the remaining 'training'. The model *w *was generated based on the 'training' set and was used to make predictions for the 'blind' set. The cross-validated distance uses a sum of squared errors as the norm.

To generate a robust model, the process of splitting the binding data, optimizing λ, and generating *w *is repeated 10 times, yielding 10 models. The final model returned by the prediction method is an average of these 10 models.

### Evaluation of the prediction methods

Five-fold cross validation was used to measure prediction performances of methods. Here, a binding dataset is divided into 5 equal subsets, where one subset is labelled a testing set and the remaining 4/5 subsets a training set. A model was generated based on the training set only and used to make predictions for the testing set. This step was repeated 4 more times by rotating around the testing set label, resulting in *blind *predictions for the full dataset in the end. Performance was measured by calculating an Area-Under-Curve (AUC) of Receiver-Operating-Characteristic (ROC) curve of the blind predictions with respect to corresponding measured binding affinities. An AUC value has a range from 0.5 to 1.0, where it can be interpreted as the probability of distinguishing binders from non-binders if they were picked randomly. Thus, a value of 1.0 indicates a perfect prediction where as that of 0.5 indicates random.

## Authors' contributions

BP conceived and designed the experiments. YK and JS performed the experiments. YK, JS, CP, AS, and BP analyzed the data. JS and CP contributed reagents/materials. YK, JS, CP, AS, and BP wrote the paper. All authors read and approved the final manuscript.

## Supplementary Material

Additional file 1 **Raw binding affinity data of combinatorial peptide scanning library against the 24 MHC molecules**. Binding affinity measurements of each peptide library are shown for all 24 MHC molecules. Each row represents a peptide library, which corresponds to a fixed residue at a given position. Columns span the 24 MHC molecules. Click here for file

Additional file 2 **Peptide:MHC Binding Energy Covariance Matrix**. This covariance matrix was calculated from the raw binding in Dataset S1. The symmetric matrix has dimensions of 20 × 20. Each entry represents how two residues contribute to binding affinities with respect to each other. A positive value indicates they are alike; a negative value indicates they are different. Click here for file

Additional file 3 **Prediction performances of NetMHC, SMM, SMM^PMBEC^, and SMMBLOSUM**. This is an expanded version of benchmark results shown in the manuscript by including prediction performances of SMMBLOSUM. Click here for file
